# Supplementary figures and images for: Dach1 is essential for maintaining normal mature podocytes
Source: PLoS One. 2024 May 28;19(5):e0303910. doi: 10.1371/journal.pone.0303910 (PMC11132487; doi:10.1371/journal.pone.0303910)

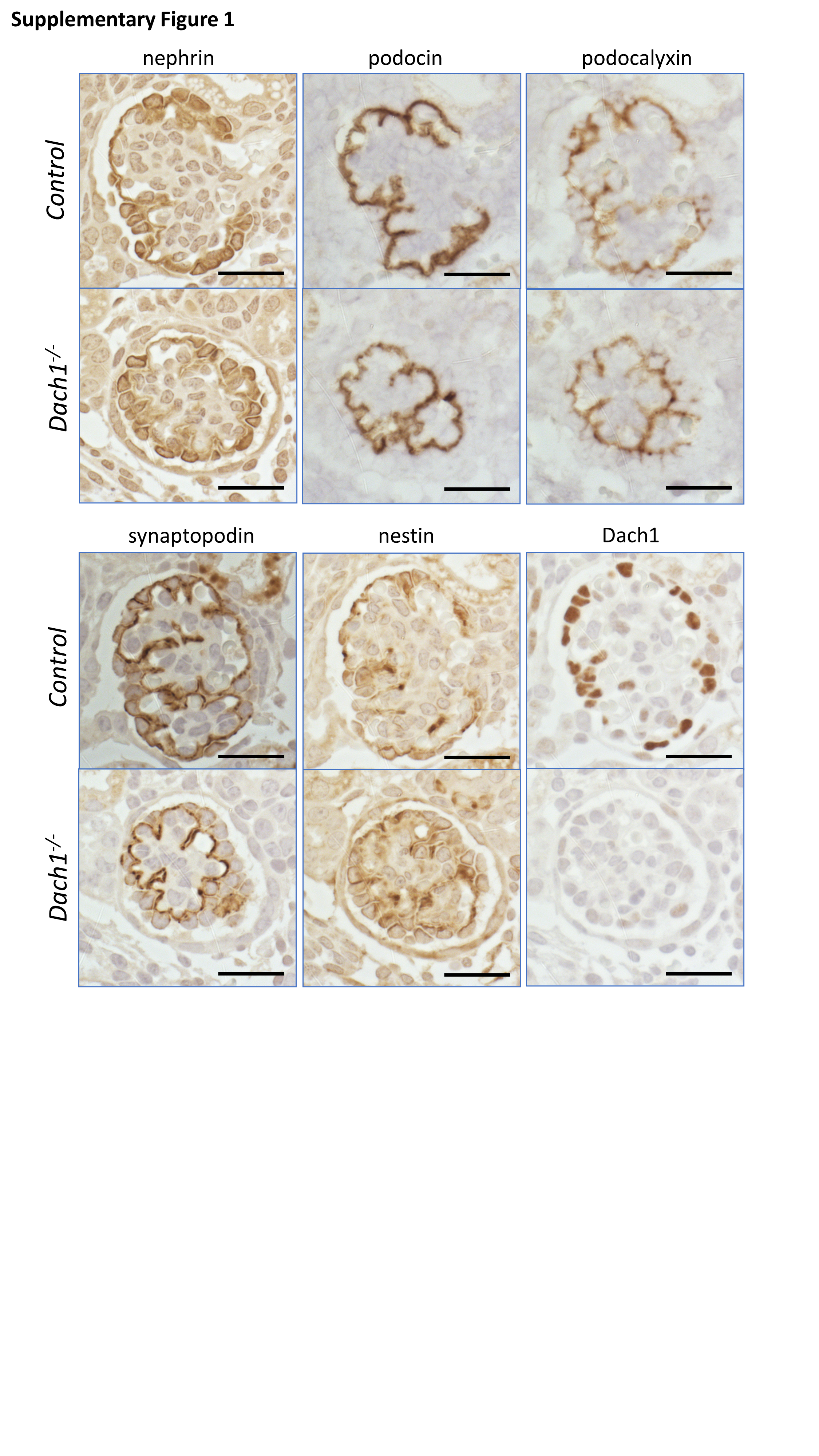

Supplement: S1 Fig — There were no differences in these podocyte proteins. Scale bar: 100 μm. (TIF) [file pone.0303910.s001.tif]

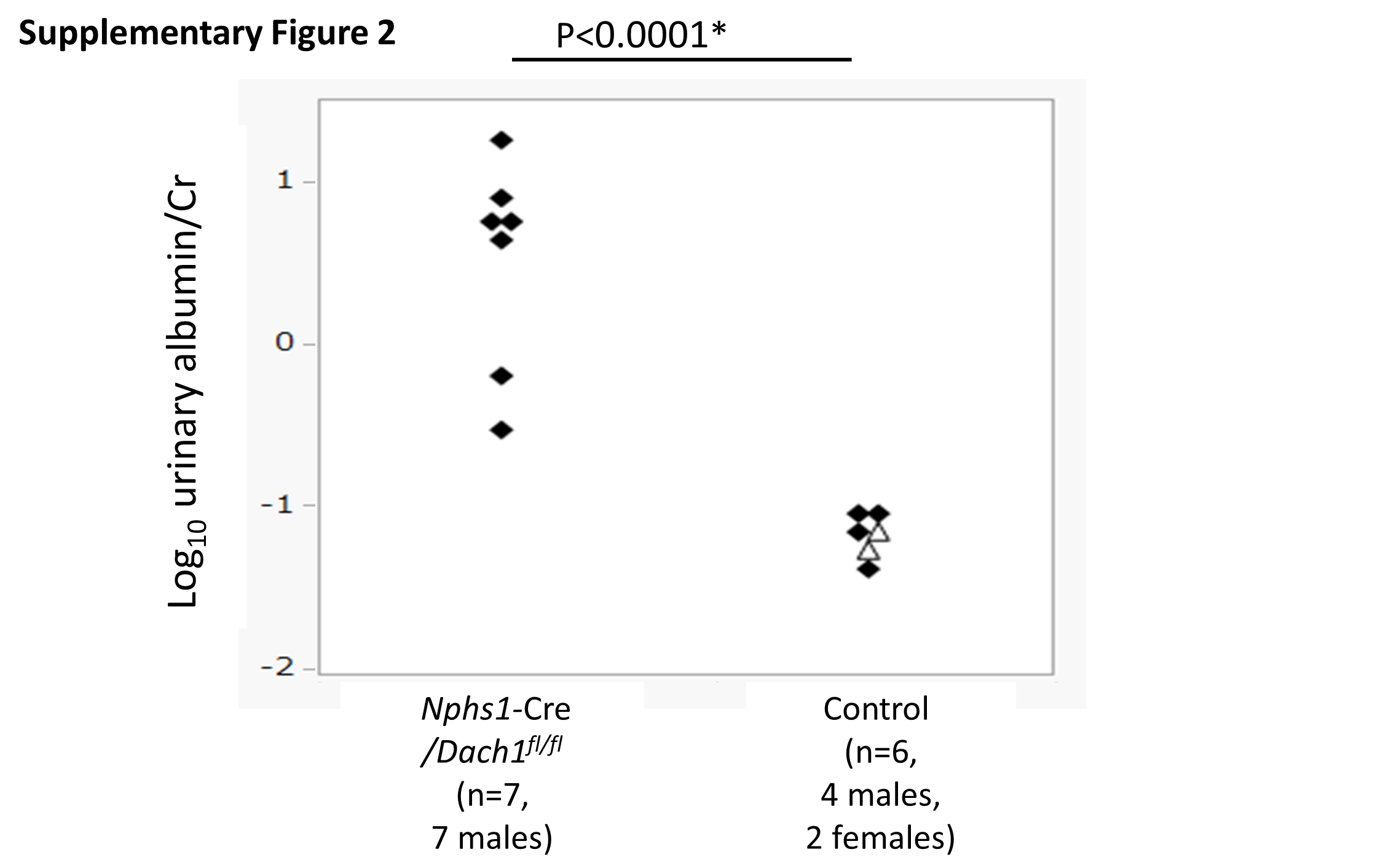

Supplement: S2 Fig — In S2 and S4 Figs, Males: black diamonds. Females: white triangles. For S4 Fig, The numbers of podocytes in intact glomeruli were counted in samples doubly stained for WT1 and synaptopodin. There was no difference in the average number of podocytes per glomerulus between Nphs1-Cre/Dach1fl/fl and control mice. Not significant (n.s.). (TIF) [file pone.0303910.s002.tif]

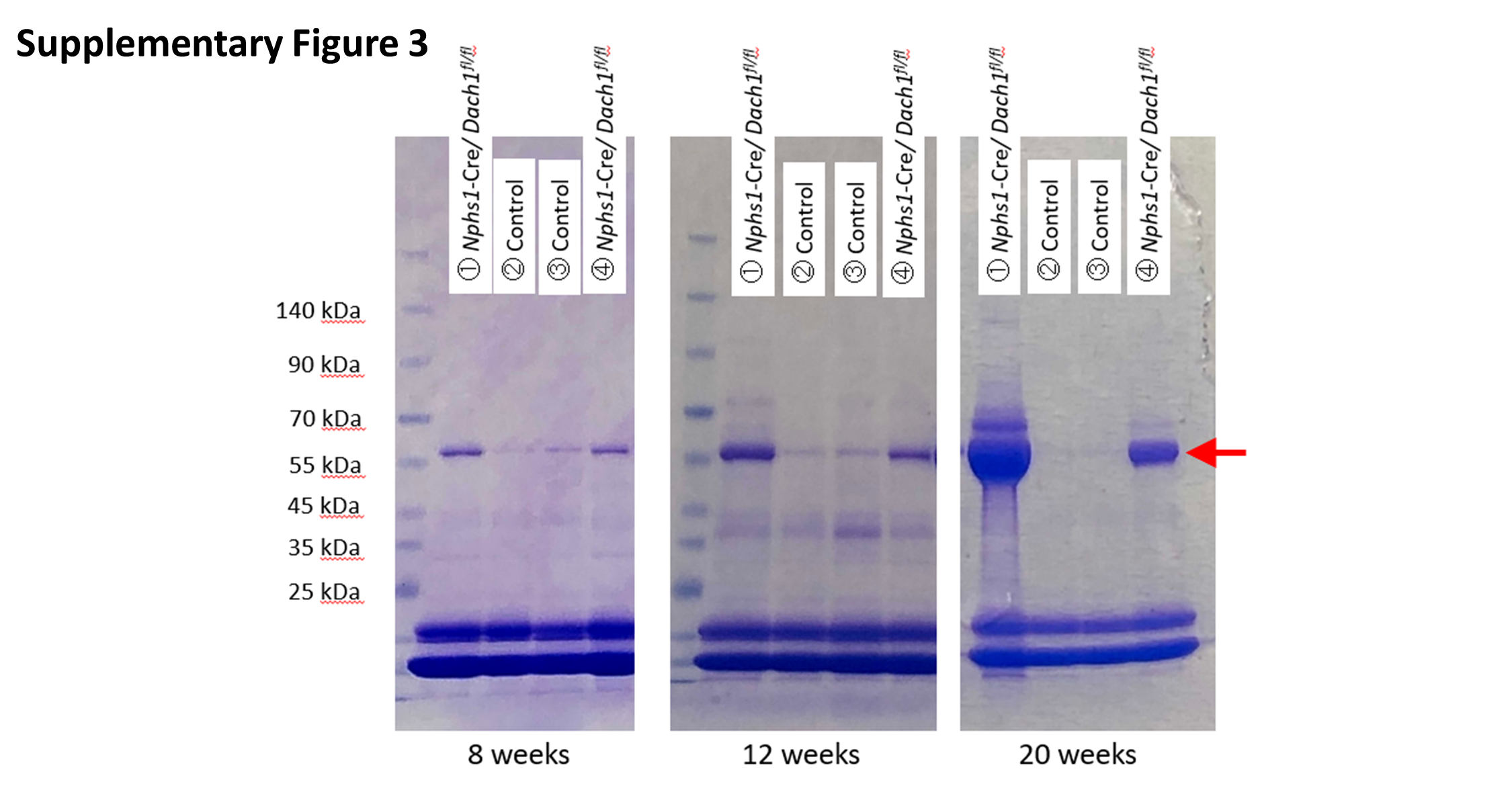

Supplement: S3 Fig — In S2 and S4 Figs, Males: black diamonds. Females: white triangles. For S4 Fig, The numbers of podocytes in intact glomeruli were counted in samples doubly stained for WT1 and synaptopodin. There was no difference in the average number of podocytes per glomerulus between Nphs1-Cre/Dach1fl/fl and control mice. Not significant (n.s.). (TIF) [file pone.0303910.s003.tif]

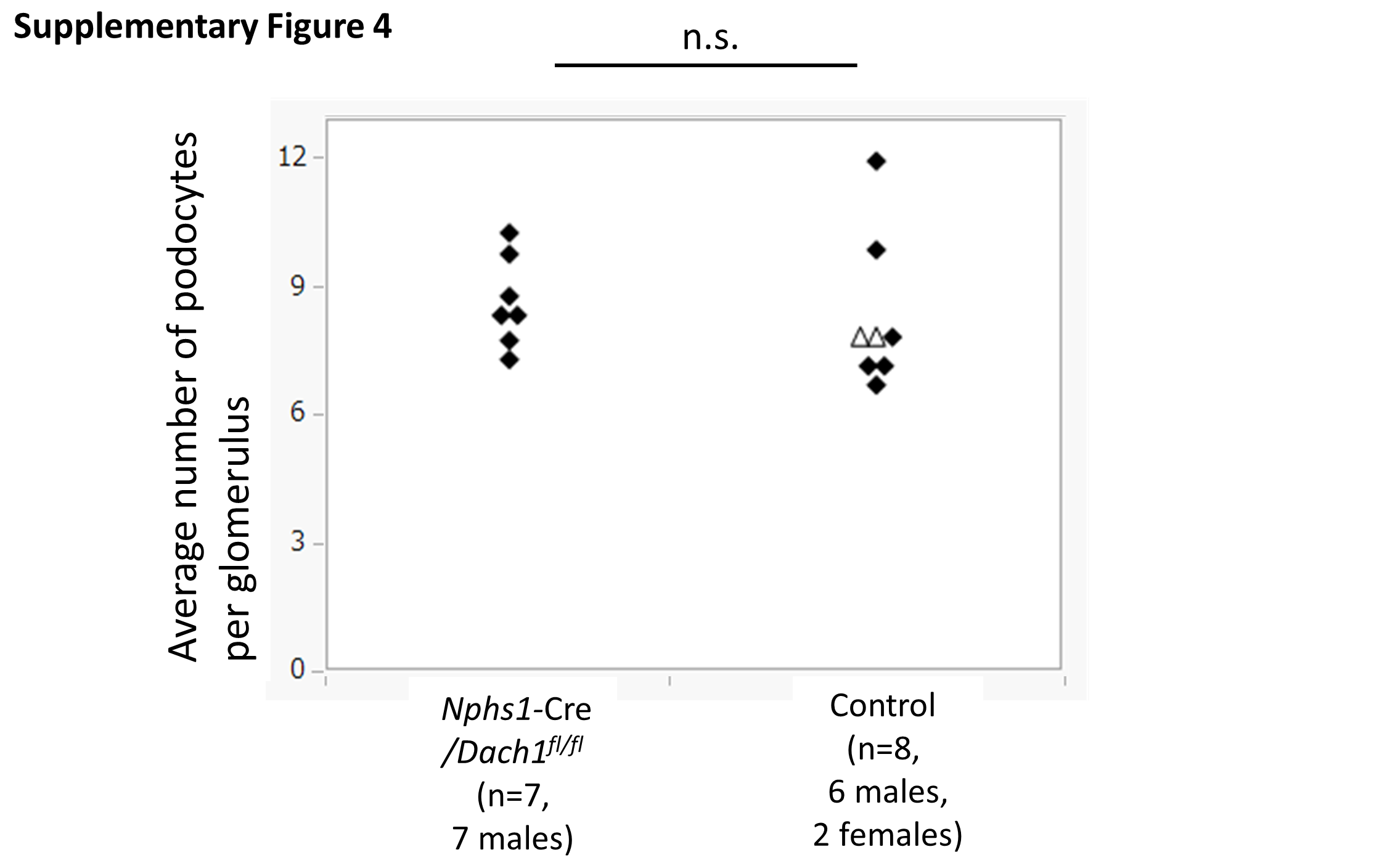

Supplement: S4 Fig — In S2 and S4 Figs, Males: black diamonds. Females: white triangles. For S4 Fig, The numbers of podocytes in intact glomeruli were counted in samples doubly stained for WT1 and synaptopodin. There was no difference in the average number of podocytes per glomerulus between Nphs1-Cre/Dach1fl/fl and control mice. Not significant (n.s.). (TIF) [file pone.0303910.s004.tif]

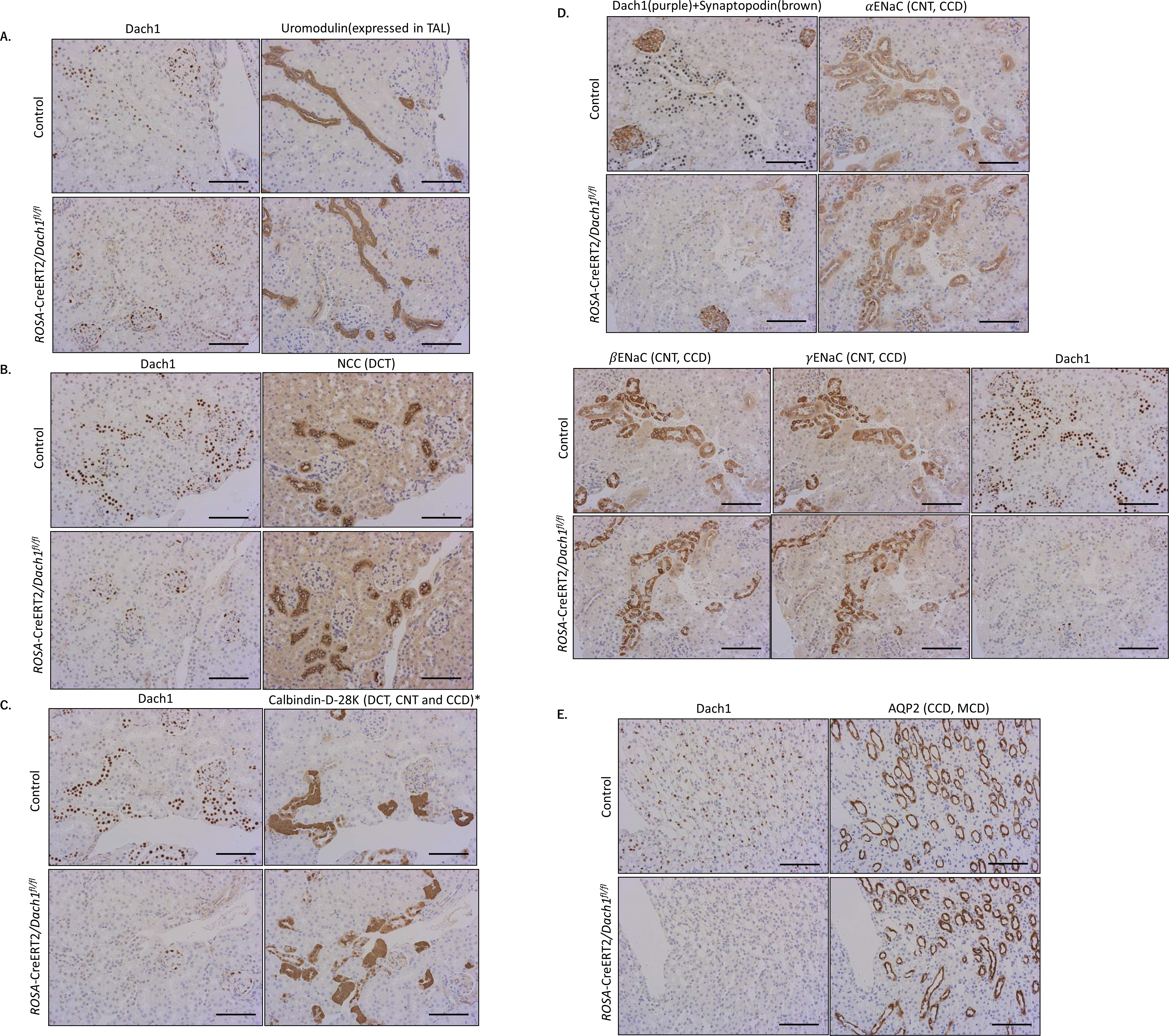

Supplement: S8 Fig — Dach1 staining was retained in glomeruli due to inefficient Cre-mediated recombination in podocytes. Although most of the tubular Dach1 staining disappeared, no abnormalities were observed in the intensity or pattern of the staining of uromodulin, NCC, calbindin-D-28K. Calbindin-D-28K is highly expressed in DCT and CNT, and weakly expressed in CCD. Information about the primary antibodies, antigen retrieval methods, and dilution ratios are listed in the S1 Table. Scale bar: 100 μm. S8 Fig. Immunostaining for Dach1 and α, β, γ ENaCs, or AQP2 in serial sections of ROSA-CreERT2/Dach1fl/fl mice. No abnormalities were observed in the intensity or pattern of the staining of α, β, γ ENaCs, or AQP2 in the kidney lacking Dach1. Information about the primary antibodies, antigen retrieval methods, and dilution ratios are listed in the S1 Table. Scale bar: 100 μm. (TIF) [file pone.0303910.s008.TIF]

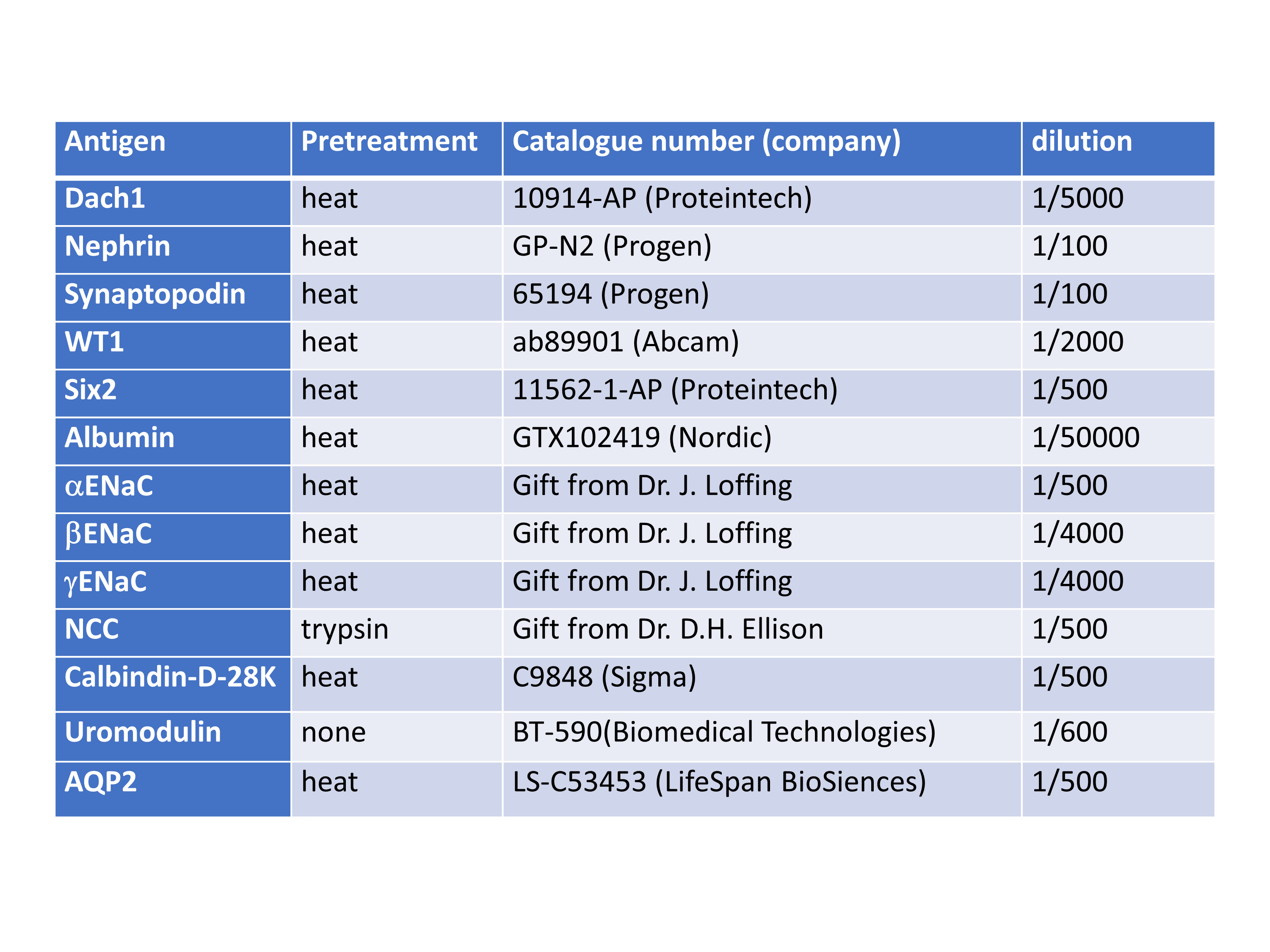

Supplement: S1 Table — (TIF) [file pone.0303910.s009.tif]

Original images for S3 Fig.

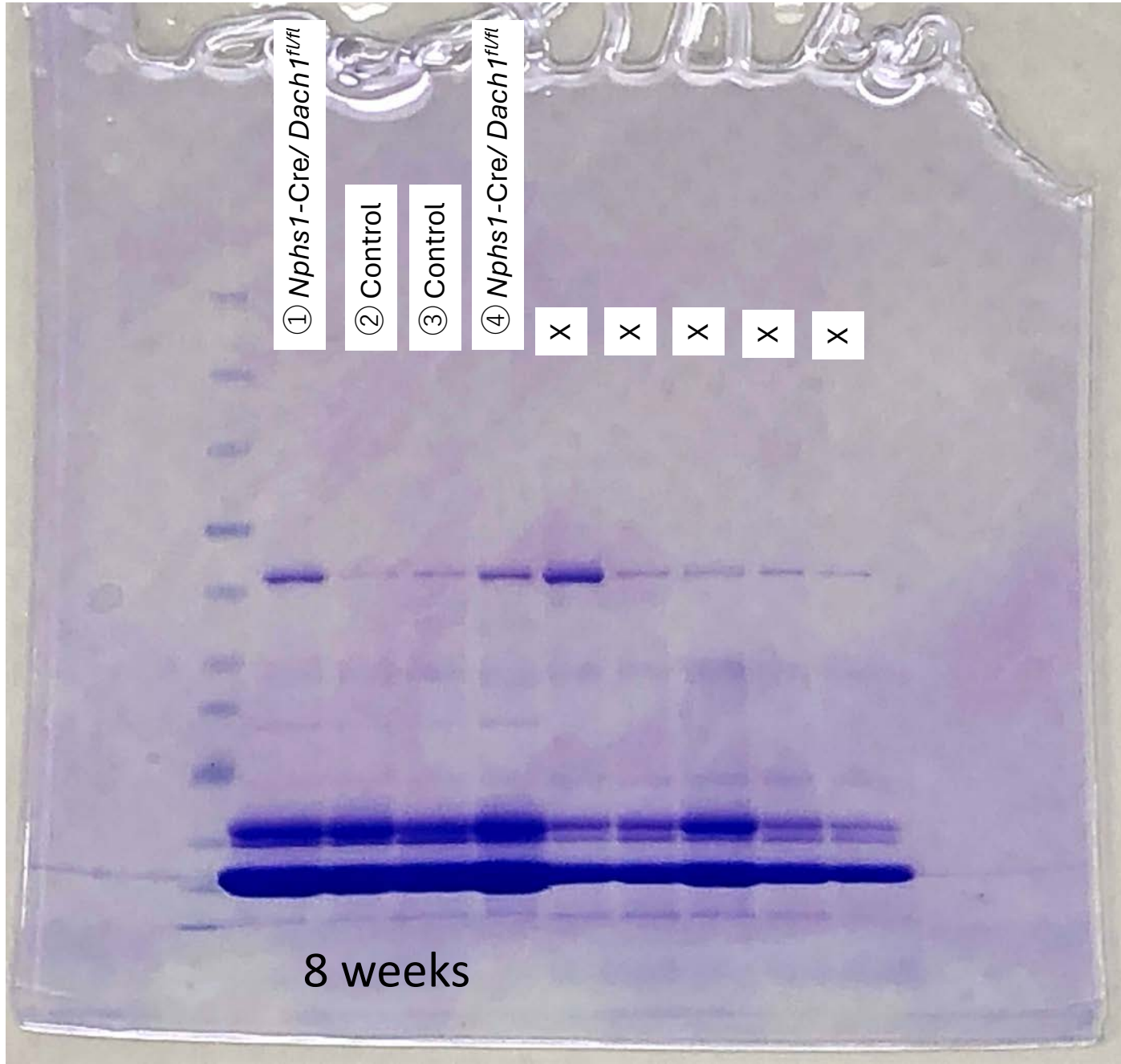

# Original images for S3 Fig.

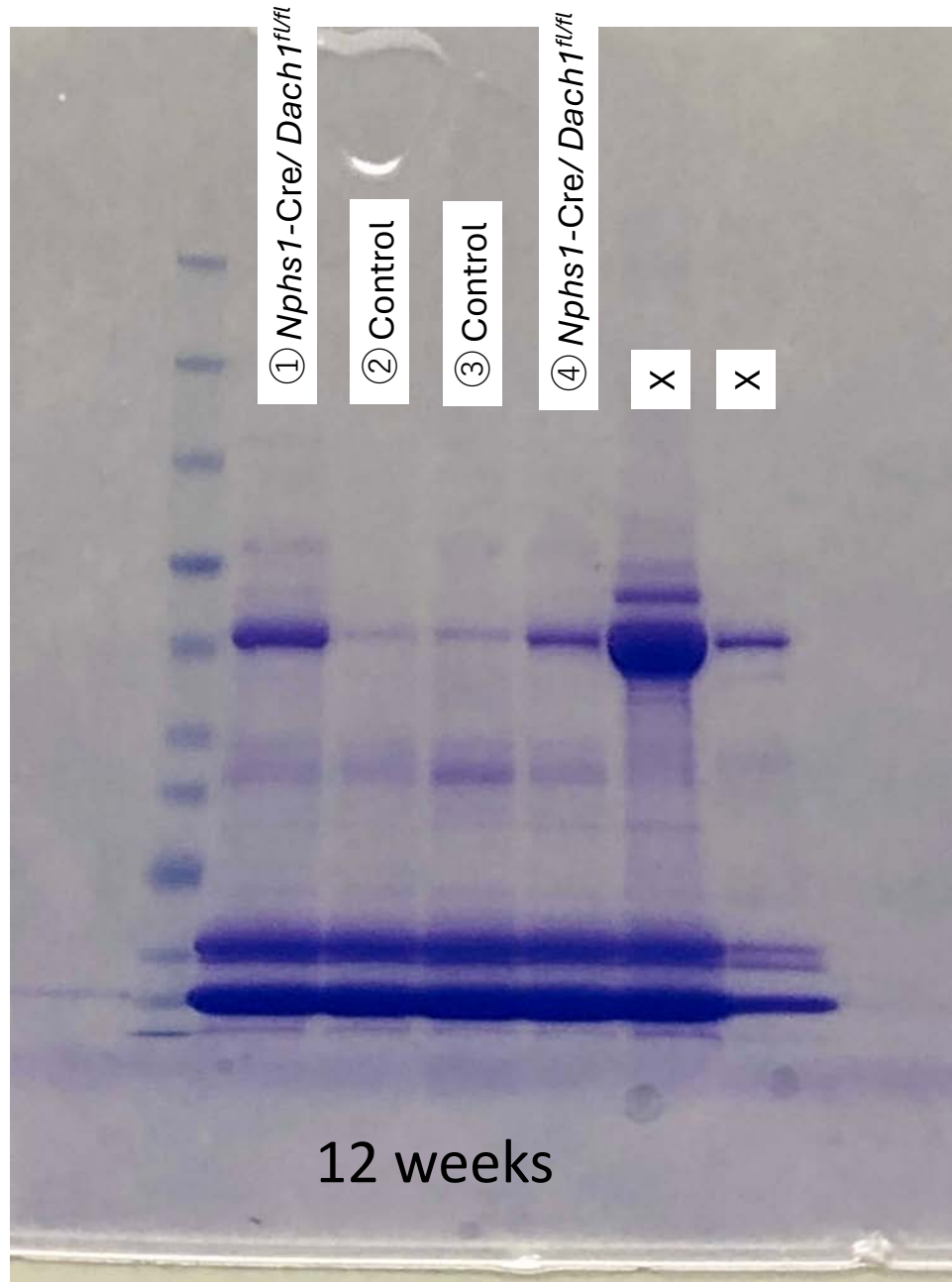

# Original images for S3 Fig.

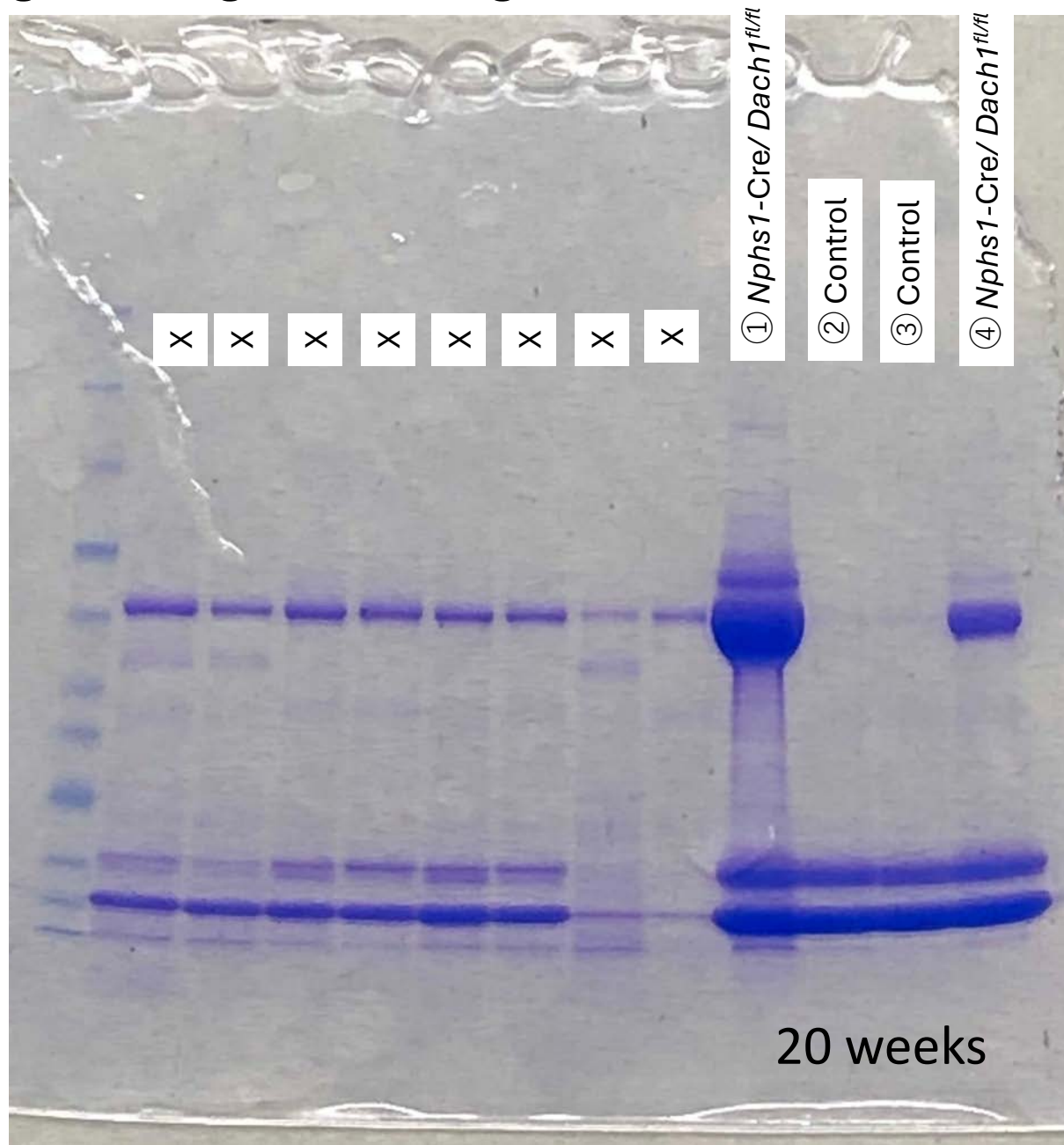

Supplement: S1 File — Original images for S3 Fig. (PDF) [file pone.0303910.s011.pdf]
